# Comprehensive meta-analysis of severe fever with thrombocytopenia syndrome virus infections in humans, vertebrate hosts and questing ticks

**DOI:** 10.1186/s13071-024-06341-2

**Published:** 2024-06-20

**Authors:** Ao-Long Xu, Han Xue, Yi Li, Xu Wang, Jin-Xin Zheng, Fu-Yan Shi, Qing-Xia Cui, Yan Lu, De-Jiao Cun, Lan-Hua Li

**Affiliations:** 1 School of Public Health, Shandong Second Medical University, Weifang, Shandong 261053 People’s Republic of China; 2 National Institute of Parasitic Diseases, Chinese Center for Disease Control and Prevention; Chinese Center for Tropical Diseases Research; National Key Laboratory of Intelligent Tracking and Forecasting for Infectious Diseases; Key Laboratory on Parasite and Vector Biology, Ministry of Health; WHO Collaborating Centre for Tropical Diseases; National Center for International Research on Tropical Diseases, Ministry of Science and Technology, Shanghai, 200025 People’s Republic of China; 3 Yunnan Center for Disease Control and Prevention, Kunming, Yunnan 650022 People’s Republic of China

**Keywords:** SFTS, Tick-borne disease, Prevalence, Meta-analysis

## Abstract

**Background:**

Severe fever with thrombocytopenia syndrome (SFTS) is an emerging tick-borne zoonosis caused by the SFTS virus (SFTSV). Understanding the prevalence of SFTSV RNA in humans, vertebrate hosts and ticks is crucial for SFTS control.

**Methods:**

A systematic review and meta-analysis were conducted to determine the prevalence of SFTSV RNA in humans, vertebrate hosts and questing ticks. Nine electronic databases were searched for relevant publications, and data on SFTSV RNA prevalence were extracted. Pooled prevalence was estimated using a random effects model. Subgroup analysis and multivariable meta-regression were performed to investigate sources of heterogeneity.

**Results:**

The pooled prevalence of SFTSV RNA in humans was 5.59% (95% confidence interval [CI] 2.78–9.15%) in those in close contact (close contacts) with infected individuals (infected cases) and 0.05% (95% CI 0.00–0.65%) in healthy individuals in endemic areas. The SFTSV infection rates in artiodactyls (5.60%; 95% CI 2.95–8.96%) and carnivores (6.34%; 95% CI 3.27–10.23%) were higher than those in rodents (0.45%; 95% CI 0.00–1.50%). Other animals, such as rabbits, hedgehogs and birds, also played significant roles in SFTSV transmission. The genus *Haemaphysalis* was the primary transmission vector, with members of *Ixodes*, *Dermacentor*, and *Amblyomma* also identified as potential vectors. The highest pooled prevalence was observed in adult ticks (1.03%; 95% CI 0.35–1.96%), followed by nymphs (0.66%; 95% CI 0.11–1.50%) and larvae (0.01%; 95% CI 0.00–0.46%). The pooled prevalence in ticks collected from endemic areas (1.86%; 95% CI 0.86–3.14%) was higher than that in ticks collected in other regions (0.41%; 95% CI 0.12–0.81%).

**Conclusions:**

Latent SFTSV infections are present in healthy individuals residing in endemic areas, and close contacts with SFTS cases are at a significantly higher risk of infection. The type of animal is linked to infection rates in vertebrate hosts, while infection rates in ticks are associated with the developmental stage. Further research is needed to investigate the impact of various environmental factors on SFTSV prevalence in vertebrate hosts and ticks.

**Graphical Abstract:**

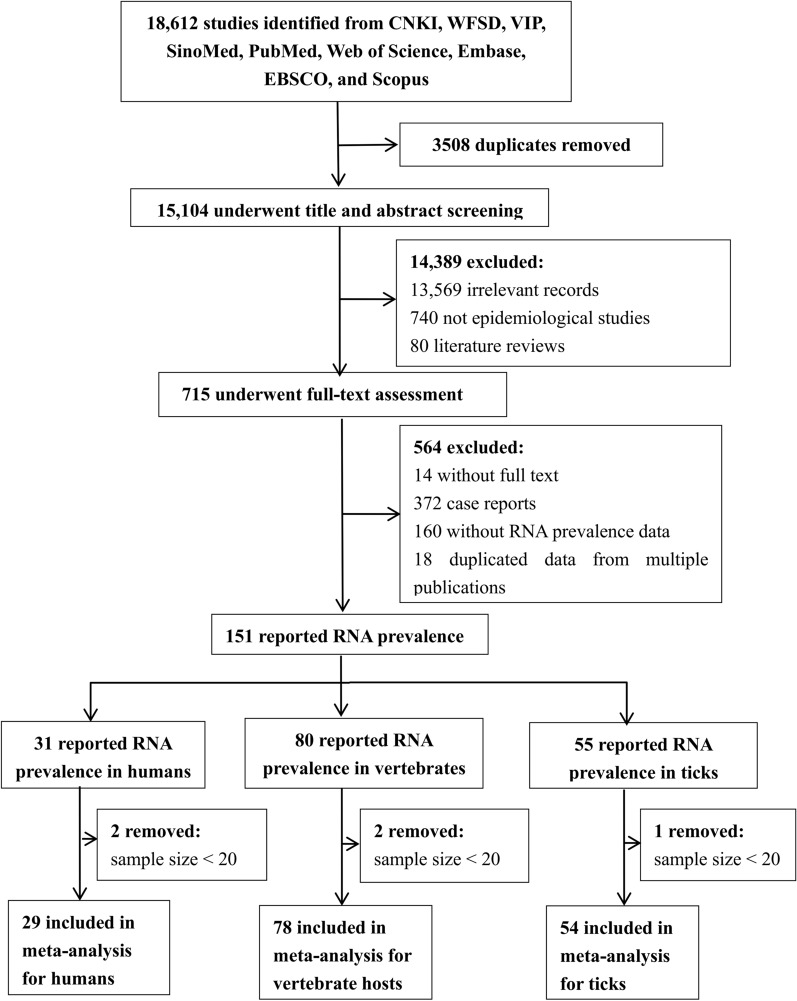

**Supplementary Information:**

The online version contains supplementary material available at 10.1186/s13071-024-06341-2.

## Background

Severe fever with thrombocytopenia syndrome (SFTS), caused by the *Dabie bandavirus* (commonly known as the SFTS virus, SFTSV), is an emerging tick-borne zoonotic disease first identified in China. SFTS typically manifests as hemorrhagic fever, thrombocytopenia, leukocytopenia, fatigue, gastrointestinal symptoms and elevated liver enzyme levels, with a case fatality rate ranging from 12% to 50% [1]. The disease can be fatal, especially in elderly patients, those with pre-existing health conditions or those who delay seeking hospital admission [2].

SFTSV belongs to the genus* Bandavirus* of the family Phenuiviridae, within the order Bunyavirales. It is found in a wide range of wild and domestic animals, including ungulates, carnivores, rodents, hedgehogs and birds. *Haemaphysalis longicornis* is the primary transmission vector for SFTSV, although other species within the *Haemaphysalis* genus, as well as certain species from the *Ixodes*, *Dermacentor*, and *Amblyomma* genera, are also implicated as potential vectors [3]. Human infections mainly occur through tick bites, although transmission can also result from contact with the blood, bodily fluids or secretions of infected individuals [4]. Since its discovery in 2009, SFTSV has been reported in various East Asian countries, including Korea, Japan, Pakistan and Vietnam [5]. However, the expanding habitats of ticks, coupled with the migratory patterns of tick-carrying birds, pose a risk for the global spread of the virus. In 2017, the WHO categorized SFTS as one of the foremost global emerging infectious diseases, with the potential to trigger a pandemic or currently lacking definitive medical resolution.

Despite extensive research on SFTSV infection rates among humans, vertebrate hosts and ticks, prevalence estimates vary significantly across studies. This variation may be attributed to differences in population subgroups, animal hosts, tick species and detection methods. Additionally, variations in animal infection rates may arise from differences in the type of specimen collected (e.g. blood or tissue). Also, the developmental stage (instars) of ticks could influence the detection rate of SFTSV in ticks. Moreover, there is a lack of a comprehensive overview of SFTSV infections in humans, vertebrate hosts and ticks. Therefore, we conducted a systematic review and meta-analysis to consolidate RNA infection rates of SFTSV across humans, animal hosts and questing ticks, and to identify the extent to which the aforementioned factors contribute to heterogeneity among studies. The findings of this study will provide crucial insights for the diagnosis, treatment, surveillance and prevention of SFTS.

## Methods

### Literature search and selection

A systematic literature search was conducted to identify studies reporting the prevalence of SFTSV RNA in humans, vertebrate hosts and ticks. The electronic databases included in the search were PubMed, Embase, Web of Science, Elton B Stephens Company (EBSCO), Scopus, Chinese National Knowledge Infrastructure (CNKI), Chinese WanFang Database (CWFD), China Biology Medicine disc (SinoMed) and Chongqing VIP Chinese Science (VIP). A full-text search was performed using the terms 'severe fever with thrombocytopenia syndrome,' 'SFTS,' 'severe fever with thrombocytopenia syndrome virus,' 'SFTSV,' 'New bunyavirus,' 'Huaiyangshan virus,' 'HYSV,' or 'Dabie bandavirus,' combined with terms like 'prevalence,' 'infection rate' or 'positive rate.' The search covered the period from the inception of the databases to 31 January 2024 and included with publications in English or Chinese.

After eliminating duplicate studies, two reviewers (AL-X and H–X) independently conducted an initial screening based on titles and abstracts. In cases of disagreement, a third reviewer (YL) was consulted to facilitate consensus. Subsequently, the same reviewers evaluated the full text of potentially eligible publications. Additionally, manual searches were conducted on the reference lists of included publications to identify any relevant studies that may have been overlooked in the electronic search. Studies were included if they could provide the prevalence of SFTSV RNA in humans, vertebrates or ticks, or provide the necessary information (numerator and denominator) for calculating RNA prevalence. Exclusion criteria encompassed reviews, letters to the editor, conference papers, case reports or case series, non-epidemiological studies, studies not reporting RNA prevalence or those with a sample size < 20.

### Data extraction and quality assessment

The following data were extracted from the eligible studies: title, first author, publication year, language, study location, type of study area (endemic or non-endemic), detection method (RT-PCR, nested RT-PCR, or real-time RT-PCR), sample size and the count of positive samples. For studies focusing on humans, information on the type of population was collected at the same time. For studies focusing on vertebrate hosts, details on the type of animal, life style (domestic or wild), taxonomic category and specimen type (blood or viscera) were collected. For studies on ticks, information on tick species, developmental stage and parasitic status (questing or infesting) was additionally gathered.

After consolidating all of the data, two reviewers (AL-X, H–X) independently evaluated the risk of bias in the included studies using the Hoy risk of bias tool. This tool comprises 10 items for assessing bias risk, with a score of 0 or 1 assigned for the absence or presence of bias in each item. An overall score ranging from 0 to 3 signifies a low risk of bias, with overall scores of 4 to 6 indicating a moderate risk of bias and of 7 to 10 indicating a high risk of bias.

### Statistical analysis

Prior to estimating the pooled infection rates, the infection rate data underwent a Freeman-Tukey double arcsine transformation. The transformed data were then utilized to estimate the pooled infection rates and their corresponding 95% confidence intervals (CI). Heterogeneity among diverse studies was assessed using the heterogeneity statistic *I*^2^ and Cochran’s Q test. A smaller *I*^2^ value indicates lower heterogeneity, with *I*^2^ < 25% denoting low heterogeneity, *I*^2^ ≥ 25 and < 50% indicating moderate heterogeneity and ≥ 50% signifying high heterogeneity. In cases where *I*^2^ ≥ 50% and the *p*-value of the Q test < 0.1, a random-effects model was employed for estimating the pooled effect size. Based on the results of the heterogeneity tests, this study ultimately employed a random-effects model for estimating the pooled infection rates.

Subgroup analysis and multivariate meta-regression analysis were conducted to explore the sources of heterogeneity and assess the impact of various subgroup variables on infection rates. *R*^2^ was utilized to ascertain the proportion of heterogeneity explained by the model. The regression model heterogeneity statistic (QM) and its *p*-value were employed to determine the statistical significance of the model in explaining heterogeneity. Additionally, the residual heterogeneity statistic (QE) and its *p*-value were utilized to evaluate whether the unexplained residual heterogeneity was statistically significant.

Funnel plots were utilized to assess the extent of publication bias, and Egger’s test was employed to evaluate the asymmetry of the funnel plot. To examine the robustness of the pooled rate estimates, sensitivity analyses were conducted. Firstly, outlier analyses were performed using Baujat plots. Studies located in the top right quadrant of the Baujat plot, or with studentized residuals > 2 in absolute value, were considered potential outliers. After removing outliers, the pooled rates were re-estimated, and the results were compared with the main outcomes to investigate the influence of outliers on the pooled rate estimates. Additionally, we recalculated the pooled infection rate after excluding data points representing the smallest 20% of the sample size, aiming to explore the impact of data points with small sample size on the pooled rate estimates.

The packages ‘meta’, ‘metafor’ and ‘weightr’ in the R 4.3.1 software package (Lucent Technologies, Murray Hill, NJ USA) were utilized to perform the meta-analysis. Unless otherwise stated, a *p*-value < 0.05 was considered to be statistically significant. This systematic review adhered to the Preferred Reporting Items for Systematic Reviews and Meta-Analyses (PRISMA) reporting guidelines and has been registered with PROSPERO under the identifier CRD42022347240.

## Results

### Literature selection and quality assessment

**Fig. 1:**
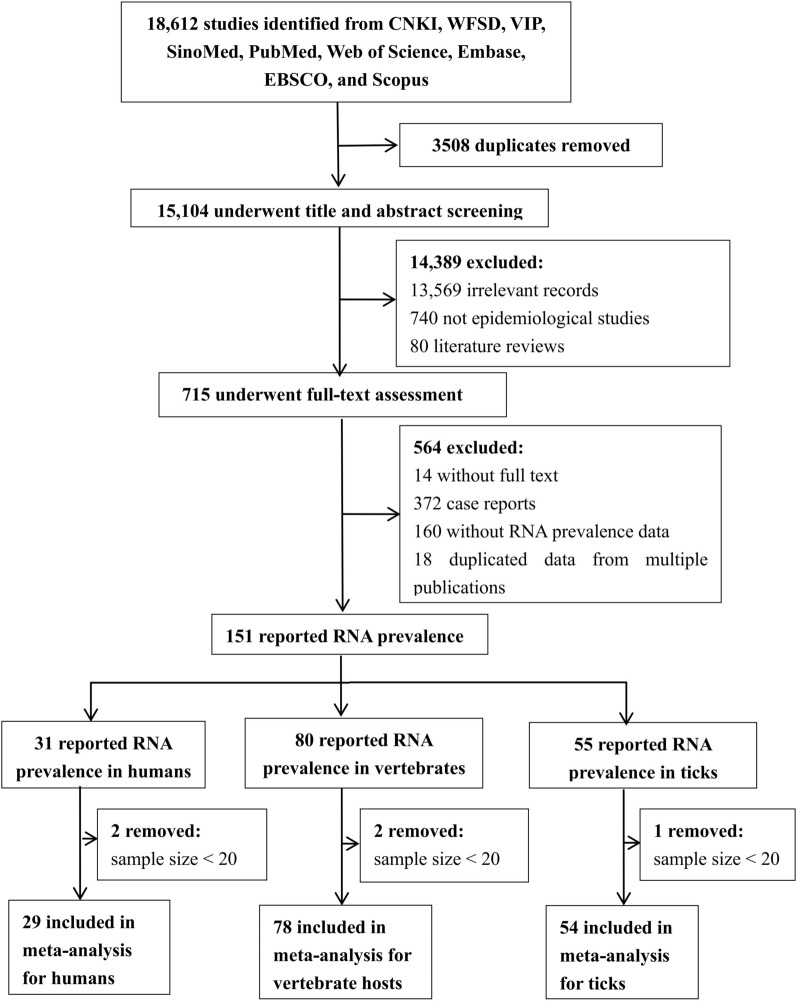
Flow diagram of study selection for the systematic review and meta-analysis

The initial literature search identified 18,612 publications. After duplicates were removed, a screening process based on titles and abstracts was conducted on 15,104 articles, resulting in 715 publications for full-text evaluation. Following a thorough review of the full-text articles, a final selection of 151 publications was included in the systematic review. Among these, 29, 78 and 54 publication were included in the meta-analysis for humans, vertebrate hosts and ticks, respectively (see Fig. 1).

The fundamental characteristics and extracted data from the included studies involving humans, vertebrate hosts or ticks are presented in Additional files 1–3: Tables S1–S3, respectively. The majority of these studies were conducted in China, followed by Korea, Japan, Thailand and Vietnam. In the assessment of bias, all studies received ratings indicating low to moderate bias levels, as detailed in Additional files 1–3: Tables S1–S3, respectively. Specifically, among the 29 studies related to humans, 11 were assessed as having low bias, while all 78 studies on vertebrate hosts and 54 studies on ticks were rated as having low bias. The predominant risk identified was the absence of random sample selection.

### Prevalence of SFTSV RNA in humans

Overall, 29 studies containing 20,412 blood samples were included in the meta-analysis to estimate the pooled prevalence of SFTSV nucleic acid in humans. Specifically, seven studies involved individuals in close contact (referred to further as close contacts) with SFTS cases, seven studies involved specific patients, 13 studies involved healthy populations from endemic areas and eight involved other healthy populations.

The heterogeneity across the studies was high across various studies (*I*^*2*^ = 91.5%; *p* < 0.0001; Table 1; forest plot is shown in Additional file 4: Figure S1). Subgroup analysis showed that type of population could explain the heterogeneity significantly (*R*^*2*^ = 58.05%; QM = 33.97; *p* < 0.0001; Table 1). The pooled prevalence of SFTSV RNA was estimated to be 5.59% (95% CI 2.78–9.15%) in close contacts, 1.83% (95% CI 0.30–4.26%) in specific patients, 0.05% (95% CI 0.00–0.65%) in healthy populations from endemic areas and 0.00% (95% CI 0.00–0.68%) in other healthy populations (see Table 1). It is worth noting that in two studies on blood donors, one study detected two SFTSV-positive samples out of 9960 blood samples [6], suggesting a potential risk of blood transmission for SFTSV (see Additional file 1: Table S1).

**Table 1:** Estimates of pooled prevalence and subgroup analysis of severe fever with thrombocytopenia syndrome virus in humans

Subgroup	No. of data points	Sample size (*n*)	No. of samples positive for SFTSV RNA	Pooled prevalence, % (95% CI)	*I*^2^ (%)	*R*^*2*^ (%)	QM (*p*)	QE (*p*)
*Type of population*	37	20,412	137	0.81 (0.14; 1.86)	91.50	58.05	33.97 (< 0.0001)	140.29 (< 0.0001)
Close contacts	9	1220	92	5.59 (2.78; 9.15)	81.40			
Specific patients	7	2909	33	1.83 (0.30; 4.26)	91.20			
Healthy population from endemic areas	13	13,510	11	0.05 (0.00; 0.65)	51.50			
Other healthy population	8	2773	1	0.00 (0.00; 0.68)	0.00			
*Detection method*						29.02	11.78 (0.0082)	172.02 (< 0.0001)
Real-time RT-PCR	25	15,917	32	0.31 (0.00; 1.17)	77.50			
RT-PCR	8	3361	27	0.76 (0.00; 2.94)	88.80			
Nested RT-PCR	1	203	1	0.49 (0.00; 7.80)	NE			
Retrospective investigation	3	931	77	8.93 (3.18; 17.03)	37.90			
*Country*						4.85	3.82 (0.1484)	403.76 (< 0.0001)
China	30	16,874	102	0.53 (0.01; 1.56)	91.20			
Korea	6	2824	35	3.35 (0.52; 8.02)	93.40			
Vietnam	1	714	0	0.00 (0.00; 5.68)	NE			

*CI* Confidence interval, *I*^2^ heterogeneity statistic,* NE* not estimated,* QE* residual heterogeneity statistic,* QM* regression model heterogeneity statistic,* RT-PCR* reverse transcription PCR,* SFTSV* severe fever with thrombocytopenia syndrome virus

The multivariable meta-regression model revealed that the population type, detection method and country together explained 60.53% of the heterogeneity (QM = 43.32; *p* < 0.0001; see Additional file 5: Table S4). Subgroup analyses also suggested a relationship between the SFTSV prevalence and the detection method (see Table 1). However, the meta-regression model indicated that the detection method did not impact the infection rate (refer to Additional file 5: Table S4).

### Prevalence of SFTSV RNA in vertebrate hosts

A total of 78 studies comprising 42,955 blood or tissue samples from vertebrate animals were included in the meta-analysis to estimate the pooled prevalence of SFTSV RNA in vertebrate hosts. The infection rates varied significantly across studies, ranging from 0.00% to 60.00% (see Additional file 2: Table S2). The pooled prevalence in vertebrate hosts was estimated to be 2.53% (95% CI 1.52–3.76%), with an *I*^2^ value of 96.20% (see Table 2; forest plot in Additional file 6: Figure S2).

**Table 2:** Estimates of pooled prevalence and subgroup analysis of severe fever with thrombocytopenia syndrome virus in vertebrate hosts

Subgroup	No. of data points	Sample size (*n*)	No. of samples positive for SFTSV RNA	Pooled prevalence, % (95% CI)	*I*^2^, %	*R*^2^, %	QM (*p*)	QE (*p*)
*Type of study area*	119	42,955	1053	2.53 (1.52:3.76)	96.20	0.00	0.17 (0.6789)	2889.66 (< 0.0001)
Endemic area	46	11,122	387	2.24 (0.80;4.23)	92.60			
Other area	73	31,833	666	2.73 (1.42;4.38)	96.80			
*Type of animal*						16.15	26.26 (0.0009)	1689.32 (< 0.0001)
Artiodactyls	27	7852	513	5.60 (2.95;8.96)	95.80			
Carnivores	23	3217	302	6.34 (3.27;10.23)	95.60			
Rodents	48	28,946	134	0.45 (0.00;1.50)	89.90			
Insectivores	5	336	5	0.51 (0.00;6.59)	0.00			
Lagomorph	2	98	16	8.56 (0.00;27.69)	91.50			
Erinaceomorpha	4	293	14	3.46 (0.00;13.07)	42.50			
Aves	8	1778	66	2.75 (0.09;7.95)	91.90			
Chiropters	1	178	0	0.00 (0.00;11.96)	NE			
Unspecified mammals	1	257	3	1.17 (0.00;17.47)	NE			
*Lifestyle of animal*						17.36	22.70 (< 0.0001)	1973.07 (< 0.0001)
Wild	38	9650	708	6.52 (4.07; 9.45)	96.30			
Domestic	78	32,996	294	0.99 (0.27; 2.01)	91.90			
Not specified	3	309	51	11.53 (1.91; 26.81)	87.00			
Specimen						6.11	8.48 (0.0144)	121.54(< 0.0001)
Blood	59	13,037	798	4.39 (2.63;6.52)	95.30			
Tissue	51	27,523	231	1.09 (0.17;2.53)	93.80			
Not specified	9	2395	24	1.32 (0.00;5.46)	89.70			
*Detection method*						0.00	0.54(0.7637)	2955.32(< 0.0001)
Real-time RT-PCR	78	35,686	836	2.52 (1.31;4.06)	97.10			
Nested RT-PCR	24	5113	183	3.24 (0.97;6.54)	87.80			
RT-PCR	17	2156	34	1.68 (0.01;5.07)	82.90			
*Country*						0.00	0.04 (0.9981)	2984.99(< 0.0001)
China	93	37,658	877	2.54 (1.38;3.97)	96.70			
Korea	17	4103	124	2.69 (0.43;6.39)	82.60			
Japan	8	1144	51	2.26 (0.00;7.81)	94.80			
Thailand	1	50	1	0.02 (0.00;26.00)	NE			

*CI* Confidence interval,* I*^2^ heterogeneity statistic,* NE* not estimated,* QE* residual heterogeneity statistic,* QM* regression model heterogeneity statistic,* RT-PCR* reverse transcription PCR,* SFTSV* severe fever with thrombocytopenia syndrome virus

The type of animal could explain the heterogeneity significantly (*R*^2^ = 16.15%; QM = 26.26; *p* = 0.0009; see Table 2). SFTSV infections have been reported in a variety of animals, with the investigations on vertebrate hosts primarily focused on artiodactyls, carnivores and rodents, with artiodactyls (5.60%; 95% CI 2.95–8.96%) and carnivores (6.34%; 95% CI 3.27–10.23%) showing higher pooled infection rates compared to rodents (0.45%; 95% CI 0.00–1.50%). Other animals, including insectivores, Lagomorph, Erinaceomorpha and Aves, also played significant roles in the transmission of SFTSV. The pooled prevalence of SFTSV RNA was estimated to be 0.51% (95% CI 0.00–6.59%) for insectivores, 8.56% (95% CI 0.00–27.69%) for Lagomorph, 3.46% (95% CI 0.00–13.07%) for Erinaceomorpha and 2.75% (95% CI 0.09–7.95%) for Aves.

Subgroup analyses indicated that the type of specimen and lifestyle of the animal were also associated with infection rates in vertebrate hosts. Blood specimens were found to have a higher infection rate (4.39%; 95% CI 2.63–6.52%) than viscera specimens (1.09%; 95% CI 0.17–2.53%), and samples from domestic animals had a higher infection rate (6.52%; 95% CI 4.07–9.45%) than those from wild animals (0.99%; 95% CI 0.27–2.01%; see Table 2). However, the meta-regression model suggested that these variables did not influence the infection rates (Additional file 7: Table S5), primarily because viscera specimens mainly came from rodents, which had lower infection rates compared to other animals. Lifestyle was also associated with the type of animal (see Additional file 3: Table S2).

### Prevalence of SFTSV RNA in ticks

A total of 55 studies involving 193,461 tick samples were included in the meta-analysis to estimate the pooled RNA prevalence of SFTSV in questing ticks. The infection rates in tick samples ranged from 0.00% to 31.94% (see Additional file 3: Table S3). While most of the infections in questing ticks were reported in *Haemaphysalis* spp., *Ixodes* spp., *Dermacentor* spp. and *Amblyomma* spp. were also identified as potential vectors of SFTSV. The pooled prevalence in ticks was calculated to be 0.68% (95% CI 0.34–1.10%), with an* I*^2^ value of 98.32% (see Table 3; forest plot in Additional file 8: Figure S3).

**Table 3:** Estimates of pooled prevalence and subgroup analysis of severe fever with thrombocytopenia syndrome virus in questing ticks

Subgroup	No. of data points	Sample size	No. of samples positive for SFTSV RNA	Pooled prevalence, % (95% CI)	*I*^2^, %	*R*^2^, %	QM (*p*)	QE (*p*)
*Type of study area*	145	193,461	1700	0.68 (0.34; 1.10)	98.32	6.76	10.26 (0.0014)	4427.24 (< 0.0001)
Endemic areas	38	18,623	599	1.86 (0.86; 3.14)	96.90			
Other areas	107	174,838	1101	0.41 (0.12; 0.81)	96.70			
*Developmental stages*	145					7.12	12.63 (0.0055)	4386.88 (< 0.0001)
Adult	43	28,404	422	1.03 (0.35; 1.96)	95.60			
Nymph	41	70,165	975	0.66 (0.11; 1.50)	98.40			
Larva	24	75,796	49	0.01 (0.00; 0.46)	78.10			
Unspecified	37	19,096	254	1.24 (0.45; 2.33)	95.30			
*Genus of ticks*	148					1.71	6.74 (0.2410)	4952.04 (< 0.0001)
*Haemaphysalis*	116	178,701	1601	0.82 (0.42; 1.31)	97.50			
*Ixodes*	14	4462	14	0.05 (0.00; 1.20)	71.60			
*Amblyomma*	3	167	4	1.87 (0.00; 9.05)	0.00			
*Dermacentor*	6	4539	1	0.01 (0.00; 1.32)	0.00			
*Rhipicephalus*	2	1359	0	0.00 (0.00; 3.72)	0.00			
Unspecified	7	4233	80	2.25 (0.23; 5.77)	97.90			
*Species of tick*	148					0.00	9.90 (0.9554)	4873.51 (< 0.0001)
*H. longicornis*	68	126,687	1076	0.98 (0.46; 1.65)	97.60			
*H. flava*	26	17,975	54	0.47 (0.00; 1.54)	86.20			
*H. hystricis*	4	788	2	0.00 (0.00; 2.84)	0.00			
*H. formosensis*	3	174	4	1.78 (0.00; 8.58)	61.20			
*H. concinna*	2	141	5	2.04 (0.00; 10.69)	78.80			
*H. japonica*	1	211	8	3.79 (0.00; 17.38)	NE			
*H. kitaokai*	2	42	0	0.00 (0.00; 8.32)	0.00			
*H. sinensis*	1	32	0	0.00 (0.00; 11.76)	NE			
*H. campanulate*	1	329	0	0.00 (0.00; 6.21)	NE			
*H. megaspinosa*	1	20	0	0.00 (0.00; 14.95)	NE			
*Haemaphysalis* spp.	7	32,302	452	1.83 (0.19; 4.88)	99.60			
*I. nipponensis*	7	1175	3	0.12 (0.00; 2.53)	46.40			
*I. persuleatus*	5	3015	11	0.05 (0.00; 2.33)	88.20			
*I. granulatus*	1	44	0	0.00 (0.00; 10.25)	NE			
*Ixodes spp.*	1	228	0	0.00 (0.00; 6.57)	NE			
*D. silvarum*	5	4360	1	0.02 (0.00; 1.60)	0.60			
*D. nuttalli*	1	179	0	0.00 (0.00; 6.86)	NE			
*A. testudinarium*	3	167	4	1.87 (0.00; 9.28)	0.00			
*R. microplus*	2	1359	0	0.00 (0.00; 3.96)	0.00			
Unspecified	7	4233	80	2.24 (0.19; 5.90)	97.90			
*Detection method*	145					0.00	0.30 (0.8604)	4910.48 (< 0.0001)
Real-time RT-PCR	74	85,251	1172	0.87 (0.38; 1.51)	98.00			
Nested RT-PCR	47	98,119	459	0.53 (0.07; 1.28)	96.00			
RT-PCR	24	10,091	69	0.43 (0.00; 1.61)	83.50			
*Country*	145					0.77	2.75 (0.2529)	4891.95 (< 0.0001)
China	52	28,284	375	1.15 (0.48; 2.04)	94.50			
Japan	19	2651	9	0.13 (0.00; 1.34)	36.70			
Korea	74	162,526	1316	0.59 (0.20; 1.12)	98.10			

*CI* Confidence interval,* I*^2^ heterogeneity statistic,* NE* not estimated,* QE* residual heterogeneity statistic,* QM* regression model heterogeneity statistic,* RT-PCR* reverse transcription PCR,* SFTSV* severe fever with thrombocytopenia syndrome virus

Subgroup analysis revealed that the type of study area (*R*^2^ = 6.76%; QM = 10.26; *p* = 0.0014) and the developmental stage of ticks (*R*^2^ = 7.12%; QM = 12.63; *p* = 0.0055) significantly contributed to explaining the heterogeneity, while other factors, such as the genus of tick, detection method and country did not significantly explain the heterogeneity (see Table 3). The pooled prevalence of SFTSV was higher in ticks collected from endemic areas (1.86%; 95% CI 0.86–3.14%) than in ticks collected in other areas (0.41%; 95% CI 0.12–0.81%). When categorized by developmental stage, the pooled prevalence of SFTSV was 1.03% (95% CI 0.35–1.96%) in adults, 0.66% (95% CI 0.11–1.50%) in nymphs and 0.01% (95% CI 0.00–0.46%) in larvae. The multivariable meta-regression model indicated that the type of study area, genus of tick, life stage, detection method and country collectively accounted for 12.90% of the total heterogeneity (QM = 31.78; *p* < 0.0043; see Additional file 9: Table S6).

When separately analyzing the data for the two most common transmission vectors, namely *Haemaphysalis longicornis* and *Haemaphysalis flava*, variations in infection rates across different life stages were also observed (*p* = 0.0422): the highest infection rate occurred among adults (1.32%; 95% CI 0.47–2.50%), followed by nymphs (0.58%; 95% CI 0.05–1.47%), while larvae exhibited the lowest infection rate (0.07%; 95% CI 0.00–0.85%; see Additional file 10: Table S7).

### Publication bias and sensitivity analysis

Funnel plots asymmetry (see Additional file 11: Figure S4) and the result of Egger’s test (for humans: *t* = 3.90; *p* = 0.0005; for vertebrate hosts: *t* = 5.35; *p* < 0.0001; for ticks: *t* = 3.87; *p* = 0.002) revealed the existence of publication bias. Results of sensitive analysis showed that neither outlier data point removal nor data points with small sample sizes changed the pooled nucleic acid prevalence estimate significantly (95% CI overlapped; see Additional file 12: Table S8).

## Discussion

In this study, we conducted a comprehensive systematic review and meta-analysis to provide estimates on the pooled RNA prevalence of SFTSV in humans, vertebrate hosts and questing ticks. Our findings suggest the presence of asymptomatic SFTSV infections among healthy residents, particularly in endemic regions, and individuals in close contact with SFTS cases face a significantly elevated risk of infection. Moreover, we observed an association between the type of animal and infection rates, with rodents exhibiting lower prevalence than artiodactyls and carnivores. Although we did not find significant differences in SFTSV infection rates among different tick genera or species, the developmental stage of ticks was found to influence the infection rate, with adult ticks showing the highest rate, followed by nymphs, with larvae displaying the lowest rate.

Humans primarily acquire SFTSV through tick bites, yet cases of human-to-human transmission via direct contact with patients have been documented. Notably, China has reported numerous clustered outbreaks of SFTS resulting from human-to-human transmission [4, 7], with South Korea also documenting nosocomial outbreaks among healthcare workers [8]. Our analysis of close contacts revealed a pooled infection rate of 5.59% (95% CI 2.78–9.15%), indicating a considerable risk associated with such exposures. Furthermore, the mortality rate for individuals infected through close contact with patients was found to be significantly higher than for those infected through tick bites [4]. Consequently, enhancing the management of SFTS patients to prevent unprotected contact with patients and corpses by healthcare workers, relatives, and others is strongly recommended. Chen and colleagues analyzed clustered outbreaks of SFTS reported by the National Public Health Emergency Event Surveillance System of China and observed notable variations in secondary attack rate (SAR) based on different contact modes, with a substantially higher SAR for blood contact (50.6%) compared to non-blood contact (3.0%) [4]. Therefore, it is advisable to collect information on contact modes during investigations of clustered outbreaks to determine the SAR for different contact modes.

Six studies investigated specific patients presenting with fever or other symptoms, with four studies confirming SFTSV positivity. Among these, two studies analyzed blood samples from suspected scrub typhus cases in South Korea, reporting positivity rates of 0.39% (9/2329) and 22.97% (17/74), respectively [9, 10]. Additionally, one study examined 159 outpatients with fever and hemorrhagic symptoms from hospitals in areas with a clustered outbreak, identifying five positive cases (3.14%); in another study, researchers retrospectively tested 58 samples from patients with high erythrocyte sedimentation rates and found two positive samples (3.45%) [11]. These studies highlight the importance of testing individuals with suspected symptoms in recognized endemic areas of tick-borne diseases, which can facilitate early patient identification and reduce mortality rates.

Thirteen studies investigated healthy residents from known SFTS endemic areas, with four studies detecting positive cases, resulting in a pooled positivity rate of 0.05% (95% CI 0.00–0.65%). These results indicate the presence of asymptomatic SFTSV infections among residents in endemic areas [12]. Notably, one study detected two SFTSV-positive samples out of 9960 samples from blood donors [6]. Although there are currently no reports of SFTSV transmission through transfusion, the high rate of secondary transmission via blood contact, exceeding 50%, indicates a potential risk of transmission through blood transfusion. Therefore, it is imperative to establish a cost-effective, highly sensitive testing strategy to screen for SFTSV in blood samples from donors in endemic areas to mitigate the risk of transfusion-related transmission.

SFTSV RNA has been detected in various wild and domestic animals, including artiodactyls, carnivores, rodents, insectivores, lagomorphs, erinaceomorpha, and even Aves, indicating the widespread presence of natural reservoirs for SFTSV. Of particular significance is the prevalence of SFTSV in domestic artiodactyls, such as cattle, goats, sheep and pigs, as well as in carnivorous companion animals, such as cats and dogs. The pooled RNA prevalence of SFTSV in artiodactyls and carnivores can reach up to 5.60% (95% CI 2.95–8.96) and 6.34% (95% CI 3.27–10.23), respectively (see Table 2), supporting the hypothesis that domestic animals may serve as amplifying reservoirs for SFTSV [13]. Consequently, the close interaction between humans and animals is believed to escalate the epidemic risk of SFTS, not only due to the increased likelihood of tick bites but also due to the potential for heightened contact with secretions from animals carrying SFTSV [14, 15]. Therefore, enhancing the management of domestic animals and minimizing outdoor activities for both livestock and companion animals can contribute to lowering the risk of human infection; for example, by adopting practices like feeding sheep with harvested grass instead of letting them graze freely in the wilderness, as practiced by farmers in specific regions.

The results of subgroup analysis and meta-regression indicate a correlation between the type of animal and infection rates, with rodents exhibiting lower pooled infection rates compared to artiodactyls and carnivores (Table 2), consistent with findings from other sero-prevalence studies [13]. This association between animal category and infection rates may arise from variations in SFTSV prevalence among different developmental stages of ticks and their preference for vertebrate hosts. Specifically, adult ticks are found to exhibit the highest SFTSV infection rate, followed by nymphs, with larvae showing the lowest infection rate [16]. Adult ticks are less inclined to infest smaller-bodied rodents; rather, they show a preference for larger animals such as carnivores and artiodactyls. Conversely, rodents are primarily infested by nymphs and larvae [17]. Additionally, this correlation may also be linked to the susceptibility of vertebrate hosts to SFTSV. However, only a limited number of studies have investigated the susceptibility of various animals to SFTSV [18–20], and it remains unknown whether prolonged or persistent infection can occur in vertebrate animals infected with SFTSV. Therefore, further research is needed to investigate the role of different vertebrate hosts in the transmission of SFTSV.

The Asian longhorned tick, *H. longicornis*, has been identified as the primary transmission vector of SFTSV [21]. However, SFTSV RNA has also been detected in many other tick species, including *Haemaphysalis flava*, *H. hystricis*, *H. formosensis*, *H. concinna*, *H. japonica*, *Ixodes nipponensis*, *I. persuleatus*, *Dermacentor silvarum*, and *Amblyomma testudinarium* (Table 2). *Haemaphysalis longicornis* is endemic to the Asia–Pacific region where it exhibits a wide host range, including various wild and domestic mammalian and avian species. Notably, this tick species possesses the ability to reproduce parthenogenetically and thrive in diverse environmental conditions [22]. Recently, the distribution range of *H. longicornis* has expanded [23], with reports of its presence in the USA, where it appears to be rapidly spreading [24]. Additionally, there have been reports of human bites by *H. longicornis* in the USA [25]. Given that birds can serve as reservoirs for SFTSV, coupled with the fact that numerous widely distributed tick species, including those from the *Ixodes*, *Dermacentor*, and *Amblyomma* genera, also act as potential transmission vectors, there exists a risk of global dissemination of the virus.

Transstadial and transovarial transmission are recognized as potential mechanisms for the maintenance of SFTSV, as all unfed tick life stages have been found to test positive for the virus. Our study further elucidates that the infection rate is influenced by the life stage of ticks, with larval ticks exhibiting a significantly lower infection rate compared to nymphs and adult ticks. This observation suggests that the efficiency of transovarial transmission for SFTSV may be lower than that of transstadial transmission in nature. The simultaneous presence of two transmission modes, transstadial transmission and transovarial transmission, could potentially heighten the risk of SFTSV spread. For example, the introduction of just one adult tick of parthenogenetic *H. longicornis* carrying SFTSV in a specific region might facilitate the virus’s entry into the area due to the possibility of transovarial transmission.

Human cases of SFTS exhibit significant spatial clustering, with a higher number of cases occurring in mountainous or hilly regions [26]. This clustering is typically attributed to the elevated tick density in these regions. However, there is limited research on how environmental factors influence pathogen infection rates in ticks. Previous studies have also shown that environmental factors not only affect tick abundance but also impact the infection rate of *Borrelia burgdorferi* in ticks [27, 28]. Similarly, our study suggests that the infection rate of SFTSV in ticks is associated with the type of study area, with higher infection rates in endemic areas compared to non-endemic areas. Therefore, further research is essential to investigate the infection rates of SFTSV and other pathogens in ticks across various habitats, with the aim to understand the impact of environmental factors on pathogen infection rates in ticks.

This study has several limitations. First, there is significant heterogeneity across studies, and the subgroup variables included in the meta-analysis of vertebrate hosts and ticks can only explain a small portion of this heterogeneity, making it challenging to effectively analyze the factors influencing infection rates. Therefore, it is recommended to record environmental characteristics during prevalence surveys to better understand the factors affecting infection rates. Secondly, the number of studies included in this research is limited, and they are unevenly distributed across different regions, posing challenges in obtaining representative estimates of infection rates for various areas. Additionally, due to language barriers, this study did not include literature in languages other than English and Chinese. Lastly, similar to meta-analyses on other tick-borne diseases, this study exhibits significant publication bias, which may distort the estimated prevalence rates, requiring a cautious interpretation of the research findings [29]. Despite these limitations, this study provides a comprehensive overview of SFTSV infections in different populations, vertebrate hosts and ticks. Our findings reveal the presence of latent SFTSV infections in healthy individuals residing in endemic regions, while close contacts of SFTS cases are at a notably elevated risk of infection. Moreover, our results suggest a relationship between the type of animal and infection rates in vertebrate hosts, while infection rates in ticks are influenced by their developmental stage.

## Conclusions

In conclusion, our study highlights several key findings regarding the prevalence and transmission of SFTSV. First, we observed latent SFTSV infections among healthy individuals residing in endemic areas, including blood donors, underscoring the potential for transfusion transmission within these regions. Moreover, individuals in close contact with SFTS cases face a significantly elevated risk of infection, emphasizing the importance of implementing preventive measures in healthcare settings and among caregivers. However, it is essential to supplement these findings with further evidence of infection, such as serology or detection of live virus, to conclusively establish the risk to in-contact patients or recipients of blood products, as our study solely focuses on RT-PCR results.

Second, our analysis revealed a wide range of animal hosts and tick species involved in the transmission cycle of SFTSV. We observed a correlation between the type of animal host and infection rates, with certain species, particularly domestic artiodactyls and carnivores, exhibiting higher infection rates. Additionally, infection rates in ticks were associated with their developmental stage, with adult ticks showing the highest prevalence. However, most of the variations in infection rates among vertebrates and ticks across different studies could not be explained by known factors. Therefore, our study emphasizes the necessity for further research to investigate the impact of various environmental factors on SFTSV prevalence in both vertebrate hosts and ticks. Understanding how environmental conditions influence the transmission dynamics of SFTSV could facilitate the development of more targeted and effective control strategies.

In summary, this study enhances our comprehension of SFTS epidemiology by consolidating available evidence concerning RNA prevalence across various human populations, vertebrate hosts and vectors. However, additional research is warranted to provide further evidence on in-contact transmission or transfusion transmission. Additionally, investigating the influence of environmental factors on the variation of SFTSV prevalence in animals is imperative for a more comprehensive understanding.

### Supplementary Information

**Additional file 1: Table S1.** Publications reporting RNA prevalence of SFTSV in humans.

**Additional file 2: Table S2.** Publications reporting RNA prevalence of SFTSV in vertebrate hosts.

**Additional file 3: Table S3.** Publications reporting RNA prevalence of SFTSV in questing ticks.

**Additional file 4: Figure S1.** Forest plots depicting the RNA prevalence of SFTSV in humans. The numbers in square brackets correspond to the study ID in Table S1.

**Additional file 5: Table S4.** Multivariable meta-regression analysis for RNA prevalence of SFTSV in humans.

**Additional file 6: Figure S2.** Forest plots depicting the prevalence of SFTSV in vertebrate hosts. The numbers in square brackets correspond to the study ID in Table S2.

**Additional file 7: Table S5.** Multivariable meta-regression analyses for RNA prevalence of SFTSV in vertebrate hosts.

**Additional file 8: Figure S3.** Forest plots depicting the prevalence of SFTSV in ticks. The numbers in square brackets correspond to the study ID in Table S3.

**Additional file 9: Table S6.** Multivariable meta-regression analysis for RNA prevalence of SFTSV in ticks.

**Additional file 10: Table S7.** Estimate of pooled prevalence and subgroup analysis of SFTSV in *H. longicornis* and *H. flava.*

**Additional file 11: Figure S4. **Funnel plot for assessing publication bias in studies reporting RNA prevalence of SFTSV in humans (**a**), vertebrate hosts (**b**) and ticks (**c**).

**Additional file 12: Table S8.** Sensitivity analysis for the pooled prevalence estimates of SFTSV in humans, vertebrate hosts and ticks.

## Data Availability

We declare that our manuscript involved complete data and no additional data are available for current submission.
