# Supplementary figures and images for: Comprehensive meta-analysis of severe fever with thrombocytopenia syndrome virus infections in humans, vertebrate hosts and questing ticks
Source: Parasit Vectors. 2024 Jun 20;17:265. doi: 10.1186/s13071-024-06341-2 (PMC11191292; doi:10.1186/s13071-024-06341-2)

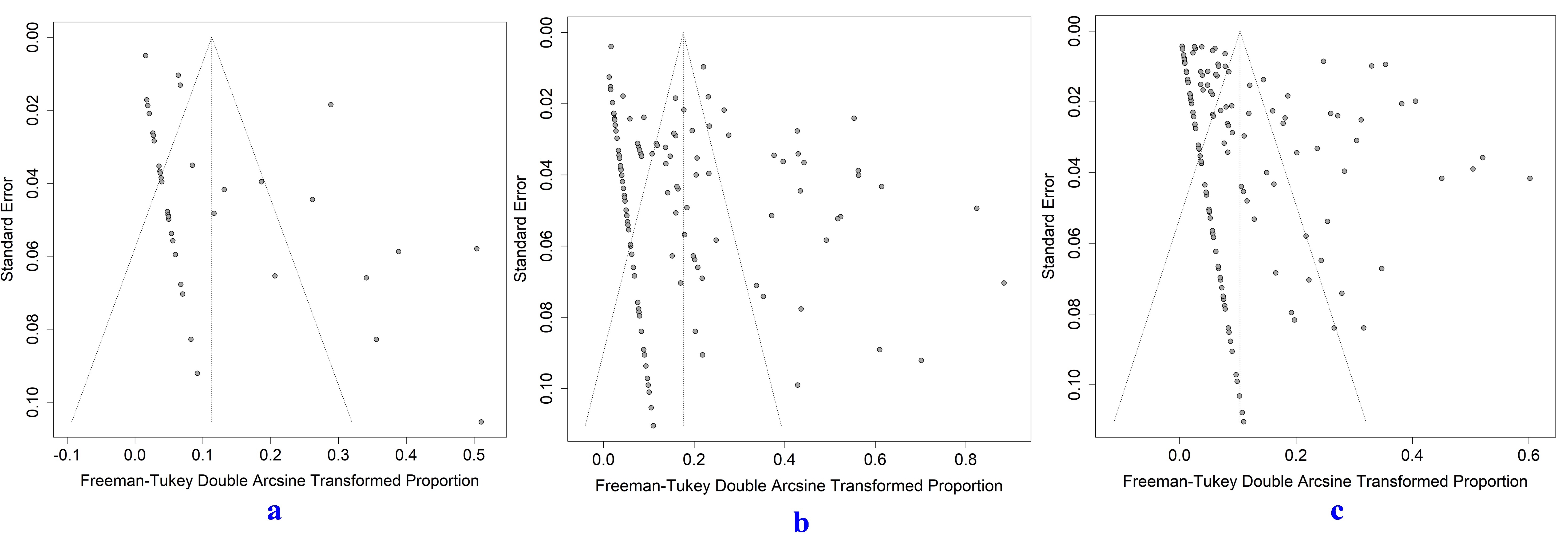

Supplement: Supplementary file 4 — Additional file 4: Figure S1. Forest plots depicting the RNA prevalence of SFTSV in humans. The numbers in square brackets correspond to the study ID in Table S1. [file 13071_2024_6341_MOESM4_ESM.jpg]
